# Raloxifene Mitigates Emotional Deficits after Mild Traumatic Brain Injury in Mice

**DOI:** 10.1089/neur.2022.0052

**Published:** 2022-11-24

**Authors:** Marcia G. Honig, Nobel A. Del Mar, Bob M. Moore, Anton Reiner

**Affiliations:** ^1^ Department of Anatomy and Neurobiology, The University of Tennessee Health Science Center, Memphis, Tennessee, USA; ^2^ Department of Ophthalmology, The University of Tennessee Health Science Center, Memphis, Tennessee, USA; ^3^ Department of Pharmaceutical Sciences, The University of Tennessee Health Science Center, Memphis, Tennessee, USA

**Keywords:** anxiety, CB2 inverse agonist therapy, depression, fear, raloxifene, traumatic brain injury

## Abstract

Persons with mild traumatic brain injury (TBI) often exhibit persistent emotional impairments, particularly depression, fearfulness, and anxiety, that significantly diminish quality of life. Studying these mood disorders in animal models of mild TBI can help provide insight into possible therapies. We have previously reported that mice show increased depression, fearfulness, and anxiety, as well as visual and motor deficits, after focal cranial blast and that treatment with the cannabinoid type 2 receptor (CB2) inverse agonist, SMM-189, reduces these deficits. We have further shown that raloxifene, which is U.S. Food and Drug Administration approved as an estrogen receptor modulator to treat osteoporosis, but also possesses CB2 inverse agonism, yields a similar benefit for visual deficits in this model of TBI. Here, we have extended our studies of raloxifene benefit and show that it similarly reverses depression, fearfulness, and anxiety after focal cranial blast TBI in mice, using standard assays of these behavioral end-points. These results indicate the potential of raloxifene in the broad rescue of deficits after mild TBI and support phase 2 efficacy testing in human clinical trials.

## Introduction

Traumatic brain injury (TBI) is highly prevalent in the United States, with an estimated 3 million to 4 million persons affected each year. Falls and motor vehicle accidents are the most common causes, and explosive blast injury is an additional contributor for members of the military.^1–4^ The vast majority (>80%) of TBIs are classified as mild^4,5^ and, despite the absence of overt brain damage, often result in impaired cognitive, emotional, sensory, and motor function. For some persons, symptoms resolve within a few weeks, but for other persons, some symptoms, typically cognitive problems (e.g., difficulty concentrating, poor memory) and mood disorders (e.g., depression and anxiety), persist for a year or even longer.^6–12^ Exaggerated fearfulness is also common,^13–15^ and persons with these persistent symptoms suffer from a diminished quality of life. Despite the wide range of impairments that can ensue after mild TBI, treatments to intervene in the injury process and thereby stem the development and/or persistence of symptoms are currently lacking.

We have previously reported that mild TBI, produced by a high-pressure air blast to one side of the mouse head, causes visual, motor, and emotional deficits along with axonal degeneration and neuron loss.^16–20^ These are attenuated by treatment with the selective cannabinoid type 2 receptor (CB2) inverse agonist, SMM-189, which targets activated microglia and biases them away from the proinflammatory M1 state toward the protective M2 state.^17,18,20,21^ Further, the reduction in optic nerve axon loss is associated with the rescue of visual deficits and appears to stem from the modulatory effects of SMM-189 on activated microglia in the optic nerve and tract.^20^ SMM-189, however, has not been tested in humans, and so we have refocused our attention on evaluating the benefit provided by raloxifene. Raloxifene is approved by the U.S. Food and Drug Administration (FDA) because of its action as a selective estrogen receptor modulator (SERM), but also acts as a CB2 receptor inverse agonist.^22,23^

We have found that raloxifene mitigates visual deficits and the associated visual system pathology resulting from mild TBI produced by focal cranial blast,^23^ or by an impact to the dorsum of the head,^24^ and by direct ocular blast.^25^ Raloxifene effectiveness for numerous end-points with these injuries strongly encourages its consideration for repurposing to treat traumatic neural injury in the human population. Here, we extend our studies of raloxifene by examining whether it also lessens depression, fear, and anxiety after focal cranial blast. Our results demonstrate a raloxifene benefit for these emotional impairments and thereby support phase 2 trial efficacy testing in humans.

## Methods

### Animals and overall experimental plan

**FIG. 1.**
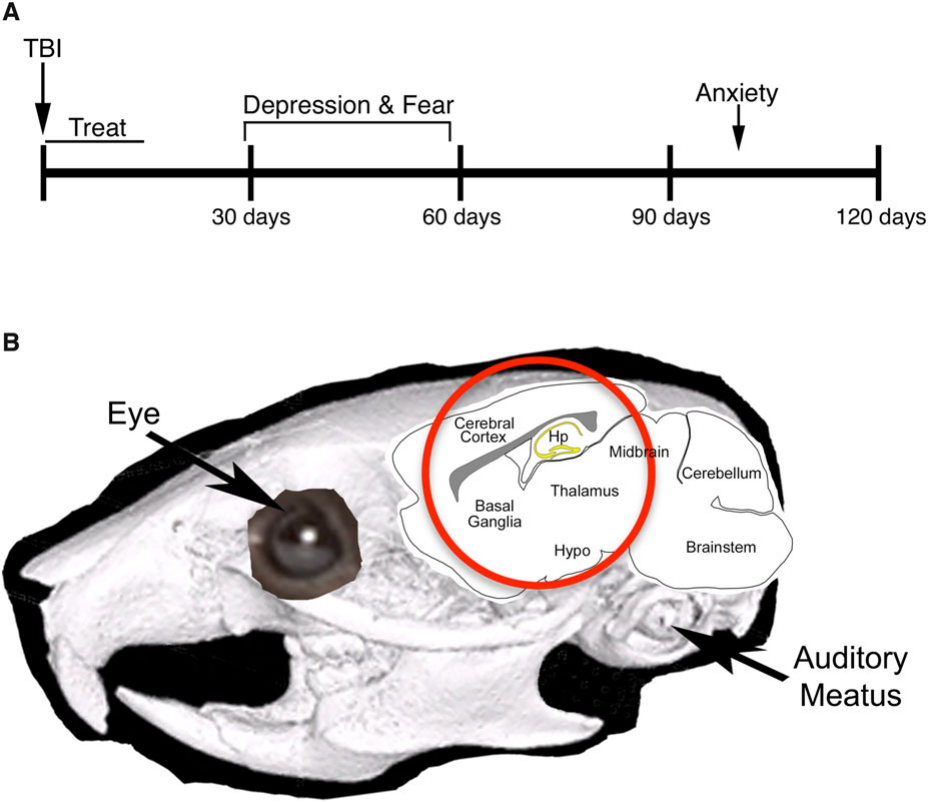
Methodology. (**A**) Timeline of experiments. Timing of blast (50 psi or sham), treatment (raloxifene or vehicle), and functional tests. Testing for depression and fear conditioning was performed 29–58 days after blast. Testing for anxiety was performed ∼100 days after blast. Four groups of mice were used for the studies: sham mice injected with vehicle, blast mice injected with vehicle, blast mice treated with 5 mg/kg of raloxifene, and blast mice treated with 10 mg/kg of raloxifene. (**B**) Image of a mouse head overlaid with a schematic of the brain showing the 7.5-mm area (red circle) targeted by the air blast (modified from Guley and colleagues^19^). Hp, hippocampus; Hypo, hypothalamus; TBI, traumatic brain injury.

Male C57BL/6 mice at 8–10 weeks of age were obtained from The Jackson Laboratory (Bar Harbor, ME). Mice were subjected to TBI at 3–4 months of age, treated for 2 weeks, and later underwent behavioral assessments (Fig. 1A). Mice tested for anxiety corresponded to some of the animals for which we previously reported visual deficits.^23^ Other cohorts of mice were used to examine depression and fear. The numbers of mice per group for each experiment are provided in the relevant figure legends. Animal studies were performed in accordance with University of Tennessee Health Science Center Institutional Animal Care and Use Committee–approved and Department of Defense–approved protocols and complied with National Institutes of Health and Society for Neuroscience guidelines.

### Blast traumatic brain injury device and blast administration

Our focal cranial blast model of mild TBI has been previously described in detail.^19^ In brief, mice were anesthetized with avertin **(**400 mg/kg, injected intraperitoneally [i.p.]**)** and inserted into protective tubing that shielded the animal, except for a 7.5-mm diameter area on the left side of the head (Fig. 1B). A single blast of 50 pounds per square inch (psi) above atmospheric pressure, which produces widespread axonal injury but no overt brain contusion,^19,26^ was delivered to the mouse head. Sham blast (0-psi) mice were handled in the same way, but with a metal plate blocking the blast wave from reaching the animal. After blast, mice were kept warm and recovered from anesthesia in 15–30 min. Acetaminophen was provided in drinking water at 1.6 mg/mL for 24 h before and after blast.

### Raloxifene and vehicle administration

Raloxifene (Sigma-Aldrich, St. Louis, MO), diluted in vehicle containing ethanol/Cremophor/0.9% saline (5:5:90), was administered at a dose of 5 or 10 mg/kg of body weight, as in our past studies.^23–25^ Mice were injected i.p. with raloxifene or vehicle, beginning 2 h after blast, and again at approximately the same time every day (±1 h) for the next 14 days (15 doses in total). For brevity, mice that received blast and vehicle will be referred to as blast-vehicle, sham mice that received vehicle as sham-vehicle or simply sham, and mice that received blast and drug as blast-ral5 or blast-ral10, depending on the dose.

### Tail suspension depression test

The tail suspension test was used to assess the characteristic of depression termed behavioral despair, as described in our past studies.^16,17^ Mice suspended by the tail eventually stop attempting to escape and become immobile, with a depressive-like state indicated by a longer duration of immobility compared to control animals. Each mouse was suspended by its tail and video recorded. Immobility was analyzed over a 5-min period using automated software (FreezeFrame; Coulbourn, Whitehall, PA).

### Auditory fear conditioning

Fear responses were examined, as previously described,^16,17,26^ using a fear-conditioning chamber (Model ENV-008; MED Associates Inc., Fairfax, VT) and automated software (FreezeFrame; Coulbourn). Mice were acclimated to the chamber for 4 min and then received five training trials, each consisting of a 12-kH tone conditioned stimulus (CS) for 30 sec, coterminating with a 0.250-sec, 0.4-mA foot shock (unconditioned stimulus; US), with 3.5 min between trials. Mice were returned to the chamber the following day, and contextual fear was assessed for 3 min. This was followed by fifteen 20-sec presentations of the CS alone, with 2 min between presentation onsets, to evaluate retention of the conditioned fear. Contextual and conditioned fear responses were measured in 20-sec time blocks.

### Light/dark box to assess anxiety

Rodent behavior in a light-dark box was first used to test the effectiveness of anxiolytics such as benzodiazepines.^27,28^ The basis of light-dark box testing is that rodents prefer dark, enclosed areas where they can hide, with more time spent in the dark compartment of the testing arena indicative of greater anxiety. As described previously,^23^ the light-dark box consisted of two equally sized compartments—an open chamber with clear walls and an enclosed chamber with black walls—connected by an opening. The test arena was covered by a black drape during the testing, to minimize outside distractions. The enclosed chamber contained a light bulb that could provide illumination at 500 or 1000 lux, and the illumination of the open chamber ranged from slightly >0 lux to a maximum of 8 lux when the enclosed chamber was at 1000 lux. Each test began with 5 min of no light in the enclosed chamber (0 lux), followed by 5 min of 500 lux and another 5 min of 1000 lux. Infrared laser beams detected the mouse location, and software measured time spent in each compartment. Given that mice are averse to bright light, they spend less time in the enclosed chamber as its illumination increases. More time spent in the enclosed arena compared to sham mice, particularly as the illumination increases, is indicative of heightened anxiety.

### Statistical analysis

Data were analyzed with one-way analysis of variance (ANOVA), followed by *post hoc* comparisons, using SPSS software (SPSS, Inc., Chicago, IL). The specific corrections for multiple comparisons are provided in the figure legends.

## Results

### Tail suspension depression test

**FIG. 2.**
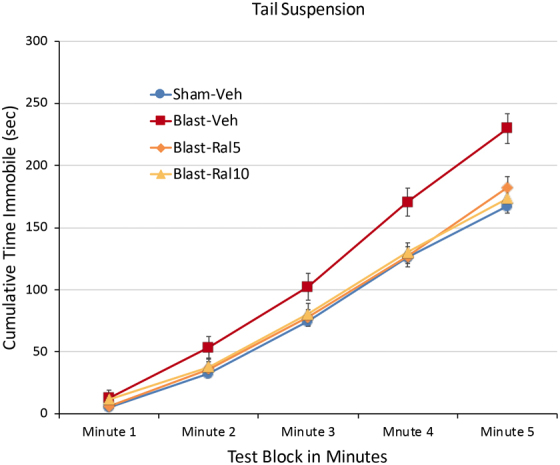
Tail suspension depression test. Data are plotted as cumulative immobility per 1-min time block. Blast-Veh mice showed a longer duration of immobility compared to Sham-Veh mice (*p* = 0.0002) over the 5-min testing period, indicative of increased depression. Immobility for Blast-Ral5 mice and Blast-Ral10 mice was significantly less than for Blast-Veh mice (*p* = 0.023 and *p* = 0.031, respectively), but not significantly different from Sham-Veh mice (*p* = 0.964 and *p* = 0.937, respectively). Thus, raloxifene normalized the depression-like state otherwise produced by focal cranial blast to sham levels. Data were analyzed with one-way ANOVA followed by Tukey's *post hoc* correction for multiple comparisons. Error bars are SEMs. Animal numbers: 24 Sham-Veh, 19 Blast-Veh, 10 Blast-Ral5, and 10 Blast-Ral10. ANOVA, analysis of variance; SEM, standard error of the mean.

As in our past studies,^16,17^ mice subjected to focal cranial blast TBI showed a longer duration of immobility, indicative of increased depression, over the 5-min testing period compared to sham-vehicle mice (*p* = 0.0002; Fig. 2). The amount of immobility for blast-ral5 and blast-ral10 mice was significantly less than for blast-vehicle mice (*p* = 0.023 and *p* = 0.031, respectively) and nearly the same as in sham-vehicle mice. Thus, both doses of raloxifene rescued the depression-like state that is otherwise produced by focal cranial blast.

### Auditory fear conditioning

#### Fear acquisition

**FIG. 3.**
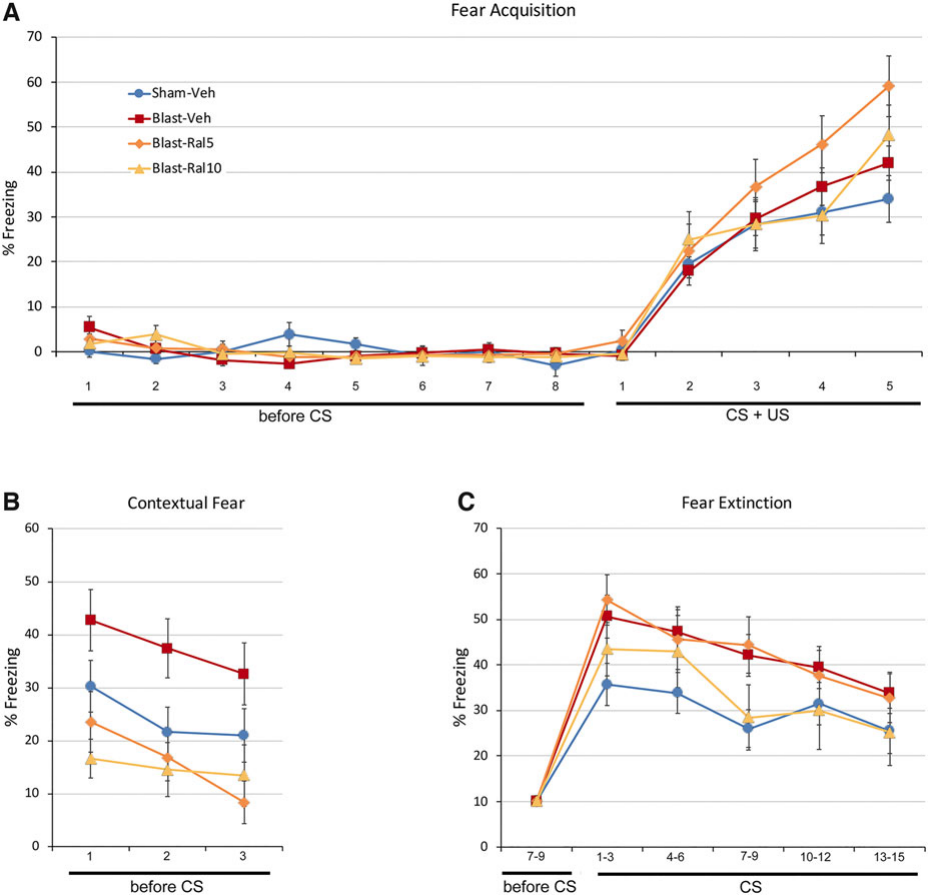
Auditory fear conditioning. Fear responses were measured by the proportion of time the animal spent freezing during each time block. (**A**) Fear acquisition. Mice were allowed to acclimatize to the chamber for 4 min (before-CS time blocks 1–8) and were then given five training trials, each consisting of an auditory cue (CS) coterminating with a foot shock (US). Freezing increased progressively with each successive training trial (CS + US time blocks 1–5). Sham-Veh and Blast-Veh mice were not significantly different (*p* = 0.527 over the last three trials). Blast-Ral10 mice were similar to Sham-Veh and Blast-Veh mice (*p* = 0.789 and *p* = 1.000, respectively). Blast-Ral5 mice, however, showed significantly more freezing over the last three trials than Sham-Veh mice (*p* = 0.005), indicative of enhanced fear learning. (**B**) Contextual fear. The next day, contextual fear was assessed for 3 min after mice were returned to the chamber, before CS-only presentations. Data are shown for the first three 20-sec time blocks. Freezing was significantly greater for Blast-Veh mice than for Sham-Veh mice (*p* = 0.005). Freezing scores for Blast-Ral5 and Blast-Ral10 mice were significantly less than for Blast-Veh mice (*p* = 0.0003 and *p* = 0.0001, respectively) and not significantly different than for Sham-Veh mice (*p* = 0.407 and *p* = 0.268, respectively). Thus, contextual fear was enhanced after focal cranial blast, and both doses of raloxifene normalized this increase. (**C**) Fear extinction. Mice received fifteen 20-sec presentations of the CS, with 2 min between presentations. Freezing values were normalized for all test blocks for each mouse so that the averages for the three 20-sec time blocks preceding the first CS presentation were 10%. Data shown are the average for three successive trials. Freezing across the 15 CS presentations was significantly greater for Blast-Veh mice than for Sham-Veh mice (*p* = 0.0002), indicating increased conditioned fear. Freezing for Blast-Ral10 mice was not significantly different than for Sham-Veh mice (*p* = 0.807) and somewhat, although not significantly, less than for Blast-Veh mice (*p* = 0.103). By contrast, freezing for Blast-Ral5 mice was similar to that for Blast-Veh mice (*p* = 1.00) and significantly greater than for Sham-Veh mice (*p* = 0.007). Thus, the 10 mg/kg dose of raloxifene, but not the 5 mg/kg dose, prevented the increase in conditioned fear produced by focal cranial blast. Data were analyzed with one-way ANOVA followed by Tukey's *post hoc* correction for multiple comparisons. Error bars are SEMs. Animal numbers: 24 Sham-Veh, 25 Blast-Veh, 10 Blast-Ral5, and 10 Blast-Ral10. ANOVA, analysis of variance; CS, conditioned stimulus; SEM, standard error of the mean; US, unconditioned stimulus.

The duration of freezing in response to the auditory CS increased progressively over the five training trials (Fig. 3A). Sham-vehicle and blast-vehicle mice did not differ significantly over the last three trials, consistent with our previous findings.^16,17^ Blast-ral10 mice behaved similarly. Thus, neither focal cranial blast nor treatment with 10 mg/kg of raloxifene altered fear acquisition. By contrast, freezing scores across the last three acquisition trials were significantly greater for blast-ral5 mice than sham-vehicle mice (*p* = 0.005), indicating that treatment with 5 mg/kg of raloxifene after focal cranial blast somehow enhanced fear learning.

#### Contextual fear

When mice were reintroduced to the testing chamber the day after fear acquisition, blast-vehicle mice exhibited significantly more freezing over the first three trial blocks (Fig. 3B) than sham-vehicle mice (*p* = 0.005), in accord with our previous results showing that contextual fear is enhanced after focal cranial blast.^16,17^ Freezing scores for blast-ral5 and blast-ral10 mice were significantly less than for blast-vehicle mice (*p* = 0.0003 and *p* = 0.0001, respectively) and not significantly different than for sham-vehicle mice. Thus, both raloxifene doses alleviated the increase in contextual fear produced by focal cranial blast.

#### Fear retention

The day after fear acquisition, blast-vehicle mice exhibited an increase in conditioned fear (Fig. 3C), in accord with our previous results.^16,17^ Freezing scores across the 15 trial blocks during CS presentation were significantly greater for blast-vehicle mice than for sham mice (*p* = 0.0002). Raloxifene at 10 mg/kg mitigated this increase, with freezing scores for blast-ral10 mice similar to those for sham-vehicle mice and somewhat, although not significantly, less than for blast-vehicle mice. By contrast, freezing scores for blast-ral5 mice were similar to those for blast-vehicle mice and significantly greater than for sham-vehicle mice (*p* = 0.007). Thus, 5 mg/kg of raloxifene did not prevent the increase in conditioned fear produced by focal cranial blast. It is uncertain whether the increased conditioned fear in blast-ral5 mice relative to sham mice stemmed from their increased fear acquisition and/or whether the lower raloxifene dose was insufficient to ameliorate the elevation in fear retention caused by focal cranial blast.

### Anxiety in the light-dark arena

**FIG. 4.**
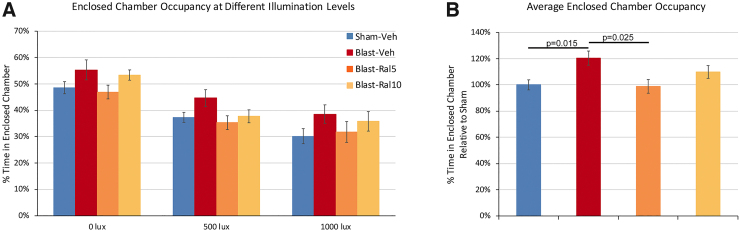
Light/dark box testing of anxiety. Mice were placed in a light-dark box with two equally sized chambers—an open clear-walled chamber kept dark and an enclosed dark-walled chamber containing a light bulb that could provide variable illumination. Each test began with 5 min of no light in the enclosed chamber, followed by 5 min of 500 lux and another 5 min of 1000 lux. (**A**) Sham-Veh mice spent roughly equal amounts of time in the open chamber and the dark enclosed chamber (51.4% and 48.6%, respectively). When the illumination of the enclosed chamber was increased to 500 lux and then to 1000 lux, Sham-Veh mouse occupancy of that chamber decreased (to 37.3% and 30.2%, respectively), because of an aversion to bright lights. Blast-Veh mice consistently spent a greater proportion of time than Sham-Veh mice in the enclosed chamber at each of the three illumination levels, suggesting heightened anxiety, although none of the differences at any given illumination level were statistically significant. Results for Blast-Ral5 mice were similar to sham, and those for Blast-Ral10 mice were intermediate between sham and Blast-Veh. (**B**) Data across illumination levels were combined for each experimental group and normalized to sham. Blast-Veh mice occupancy of the enclosed chamber was significantly greater than that for Sham-Veh mice, suggesting that Blast-Veh mice exhibit heightened anxiety. Blast-Ral5 mice spent significantly less time in the enclosed chamber than Blast-Veh mice and were similar to Sham-Veh mice (*p* = 0.998). Blast-Ral10 mice did not differ significantly from Sham-Veh mice (*p* = 0.386) or from Blast-Veh mice (*p* = 0.471). Thus, the low dose of raloxifene completely attenuated the increase in anxiety produced by focal cranial blast, and the high dose provided partial rescue. Data were analyzed with one-way ANOVA followed by Games-Howell's *post hoc* correction for multiple comparisons. Error bars are SEMs. Animal numbers: 15 Sham-Veh, 16 Blast-Veh, 17 Blast-Ral5, and 10 Blast-Ral10. ANOVA, analysis of variance; SEM, standard error of the mean.

Sham-vehicle mice spent roughly equal amounts of time in the two chambers at the start of the testing session. They decreased their occupancy of the enclosed chamber as its illumination was increased (Fig. 4A), because of the aversiveness of the increasingly brighter lights. Blast-vehicle mice consistently spent a greater proportion of their time than sham-vehicle mice in the enclosed chamber at each of the three illumination levels, although the differences were not statistically significant. However, combining the data across illumination levels (Fig. 4B) revealed that blast-vehicle mice occupancy of the enclosed chamber was significantly greater than for sham-vehicle mice (*p* = 0.014), suggesting that blast-vehicle mice exhibit heightened anxiety. Occupancy of the enclosed chamber by blast-ral5 mice was significantly less than for blast-vehicle mice (*p* = 0.025) and similar to sham-vehicle mice, showing that 5 mg/kg of raloxifene reversed the increase in anxiety produced by focal cranial blast. Blast-ral10 mice did not differ significantly from sham-vehicle mice or blast-vehicle mice, suggesting that the higher raloxifene dose partially rescued the increase in anxiety.

## Discussion

In the present study, we have extended our evidence for raloxifene benefit in mice after mild TBI produced by focal cranial blast from visual system deficits^23^ to emotional disorders. We show that mice exhibit depression, anxiety, and increased contextual and conditioned fear a few months after the injury and that raloxifene treatment reduces these behavioral impairments. Similar emotional abnormalities have been observed in rodents using other injury models^29,30^ and in humans who have experienced a mild TBI.^6–15,31^ Given that effective treatments to mitigate deficits after mild TBI are lacking, our findings support the notion that raloxifene could be repurposed for human use.

### Mild traumatic brain injury

Numerous brain regions are involved in regulating mood, including pre-frontal, insular and anterior cingulate cortex, hippocampus, several amygdala subnuclei, nucleus accumbens, and the bed nucleus of the stria terminalis.^11,32–35^ The precise brain regions that cause depression, fear, and anxiety after mild TBI, and the nature of the alterations in them, are not well understood. Thus, determining how raloxifene acts on specific regions and cells within the brain to yield functional rescue, though desirable, is beyond the scope of the current study.

Mild TBI is characterized by widespread axonal injury.^5,36,37^ For our experimental model, focal cranial blast, damaged axons are abundant in white matter tracts after a few days^19,20,23^ and degenerating axons are visualizable after a few weeks.^19^ Such axonal injury may disturb communication between (and within) brain regions that regulate emotional state and thereby lead to a disordered mood.^14,15^ Neuronal loss^18^ and altered synaptic signaling ensuing from mild TBI may similarly disrupt communication between brain regions. The reduction in axon and neuron loss with raloxifene treatment,^23^ as well as the possible restoration of normal synaptic signaling, would then lessen the amount of disruption and thereby mitigate emotional deficits.

The axonal injury occurring after mild TBI sets off a series of secondary processes, one of the more prominent being microglial activation. The initial microglial responses are typically proinflammatory and, as such, cause further damage.^38–41^ We have, in fact, found activated microglia alongside damaged axons in white matter tracts^19,20,23^ and in some brain regions^18,26^ a few days after focal cranial blast, and increased expression of proinflammatory M1 markers.^18,20,23^ Consistent with a role of activated microglia, brain levels of proinflammatory cytokines are elevated in rodent models of depression, fear, and anxiety and with depression in humans.^31,42–48^ Thus, microglial activation in mood-regulating brain regions may contribute to progression of the injury after mild TBI and exacerbate the outcome. As an aside, it should be noted that our experimental approach does not cause contusive injury to the brain, and our previous morphological studies have not revealed any obvious macrophage invasion of the brain parenchyma.

### Cannabinoid type 2 receptor inverse agonism

Microglia rapidly upregulate CB2 expression when neighboring cells are damaged, allowing drugs that bind CB2 receptors to specifically target microglia.^40,49–52^ CB2 inverse agonists stabilize CB2 receptors, which are otherwise constitutively active, in an inactive state, thereby biasing microglia away from the proinflammatory M1 state, toward the protective M2 state. CB2 inverse agonists thus derive their benefit from both types of actions.^21,53,54^ Our previous studies have demonstrated that the loss of optic nerve axons that otherwise ensues after focal cranial blast TBI is significantly reduced by 2 weeks of treatment with either raloxifene^23^ or the CB2 inverse agonist, SMM-189,^20^ suggesting that microglia can drive axonal degeneration.

In addition, SMM-189 mitigates abnormalities in oscillatory neuronal activity recorded from the hippocampus and pre-frontal cortex^55^ and rescues around half the neuron loss in cortex and striatum,^18^ supporting a role for microglia in these processes. More relevant to the work reported here, we have also shown that SMM-189 normalizes depression and contextual fear and diminishes conditioned fear after focal cranial blast.^17^ Further, SMM-189, the CB2 inverse agonist SR144528, and raloxifene yield a similar rescue of the contrast-sensitivity deficit produced by focal cranial blast, consistent with the idea that raloxifene rescue of visual deficits is attributable to effects on CB2 receptors.^17,20,,23^

### Estrogenic effects of raloxifene

Although we have previously shown that raloxifene benefit for visual contrast sensitivity does not depend on its estrogenic effects^23^ and that raloxifene modulates microglia after brain and ocular injury,^23–25^ we cannot rule out the possibility that the rescue of emotional disorders reported here is partly, or entirely, attributable to raloxifene action at estrogen receptors. Estrogen is known to exert neuroprotective effects through multiple mechanisms, including inhibiting apoptosis and decreasing neuroinflammation.^56^ Estrogen receptors are expressed by microglia, neurons, and astrocytes in diverse brain regions, including the amygdala, pre-frontal cortex, and hippocampus.^57^ Raloxifene, like other SERMS, can elicit either agonist or antagonist effects at estrogen receptors, depending on the tissue, cell type, and context.^58,59^

### Raloxifene dosing

An unresolved issue from the present study concerns differences in results for the two raloxifene doses. Raloxifene at 5 and 10 mg/kg provided similar benefit for depression and contextual fear, but the lower dose was better for anxiety, whereas the higher dose was better for conditioned fear. We previously observed dose differences in other functional and structural assessments and on the expression of M1- and M2-state microglial markers,^23–25^ suggesting that these may stem from variability in the extent and timing of microglial CB2 upregulation in different brain regions because of variation in severity of the injury in those regions. Regardless of the results in animal studies, optimizing the dosage, dosing frequency, and treatment window would be important components of any initial clinical testing. Although we typically administer raloxifene starting 2 h after injury, delaying treatment up until day 3 after focal cranial blast TBI is still effective,^23^ adding to the feasibility of raloxifene therapy. That a treatment period of only 2 weeks provides benefit for many of the end-points in the current and our previous studies^23–25^ suggests that a lengthy period of treatment would not be necessary.

### Therapeutic implications for humans

An important drawback in beginning to assess the efficacy of SMM-189 and SR144528 for mild TBI in patients is that neither has undergone phase 1 safety trials. By contrast, given that raloxifene is already FDA approved, phase 2 clinical trials could start at any time. Raloxifene has been used since the late 1990s for treating post-menopausal osteoporosis. It can be taken orally, is also safe and effective in men, and has no evident adverse hormonal side effects.^60–63^ Interestingly, daily treatment with 120 mg of raloxifene (twice the dose used for osteoporosis) improves attention and memory in schizophrenic patients,^63^ indicating sufficient brain penetration for achieving benefit. Although we have administered raloxifene at yet higher doses, those doses are known to be safe in humans.^58^

We have previously demonstrated that raloxifene improves visual outcomes in mice after mild TBI produced by focal cranial blast,^23^ mild TBI produced by an impact to the head,^24^ and after ocular blast injury.^25^ Here, we expand upon our evidence for raloxifene benefit, showing that raloxifene reduces the depression, heightened fearfulness, and anxiety ensuing from focal cranial blast TBI. Given that mild TBI in humans often leads to a similar set of persistent, debilitating symptoms, a regimen of raloxifene delivered in the early aftermath of the injury may help reduce adverse emotional outcomes, as well as visual impairments. Taken together, our findings strongly support the consideration of raloxifene for treating mild TBI in humans.

## Data Availability

The data from this present study on raloxifene are available from the investigators upon reasonable request.

## Abbreviations Used

ANOVA: analysis of variance
CB2: cannabinoid type 2 receptor
CS: conditioned stimulus
FDA: U.S. Food and Drug Administration
i.p.: intraperitoneally
psi: pounds per square inch
SERM: selective estrogen receptor modulator
TBI: traumatic brain injury
US: unconditioned stimulus
